# Alternative splicing signature of alveolar type II epithelial cells of Tibetan pigs under hypoxia-induced

**DOI:** 10.3389/fvets.2022.984703

**Published:** 2022-09-16

**Authors:** Haonan Yuan, Xuanbo Liu, Zhengwen Wang, Yue Ren, Yongqing Li, Caixia Gao, Ting Jiao, Yuan Cai, Yanan Yang, Shengguo Zhao

**Affiliations:** ^1^College of Animal Science and Technology, Gansu Agricultural University, Lanzhou, China; ^2^Academy of Agriculture and Animal Husbandry Sciences, Institute of Animal Husbandry and Veterinary Medicine, Lhasa, China; ^3^Xinjiang Academy of Animal Sciences, Xinjiang, China; ^4^State Key Laboratory of Veterinary Biotechnology, Harbin Veterinary Research Institute, Chinese Academy of Agricultural Sciences, Harbin, China; ^5^College of Grassland Science, Gansu Agricultural University, Lanzhou, China

**Keywords:** alternative splicing, hypoxia, ATII cells, swine, MAPK signaling pathway, glycolysis/gluconeogenesis

## Abstract

Alternative splicing (AS) allows the generation of multiple transcript variants from a single gene and affects biological processes by generating protein diversity in organisms. In total, 41,642 AS events corresponding to 9,924 genes were identified, and SE is the most abundant alternatively spliced type. The analysis of functional categories demonstrates that alternatively spliced differentially expressed genes (DEGs) were enriched in the MAPK signaling pathway and hypoxia-inducible factor 1 (HIF-1) signaling pathway. Proteoglycans in cancer between the normoxic (21% O_2_, TN and LN) and hypoxic (2% O_2_, TL and LL) groups, such as *SLC2A1, HK1, HK2, ENO3*, and *PFKFB3*, have the potential to rapidly proliferate alveolar type II epithelial (ATII) cells by increasing the intracellular levels of glucose and quickly divert to anabolic pathways by glycolysis intermediates under hypoxia. *ACADL, EHHADH*, and *CPT1A* undergo one or two AS types with different frequencies in ATII cells between TN and TL groups (excluding alternatively spliced DEGs shared between normoxic and hypoxic groups), and a constant supply of lipids might be obtained either from the circulation or *de novo* synthesis for better growth of ATII cells under hypoxia condition. *MCM7* and *MCM3* undergo different AS types between LN and LL groups (excluding alternatively spliced DEGs shared between normoxic and hypoxic groups), which may bind to the amino-terminal PER-SIM-ARNT domain and the carboxyl terminus of *HIF*-*1*α to maintain their stability. Overall, AS and expression levels of candidate mRNAs between Tibetan pigs and Landrace pigs revealed by RNA-seq suggest their potential involvement in the ATII cells grown under hypoxia conditions.

## Introduction

Tibetan pigs adapt well to hypoxic environments compared to other pigs, as the native breeds live in the Qinghai-Tibet Plateau (1). Studies have shown that Tibetan pigs have evolved typical characteristics to adapt to high-altitude hypoxia, especially with developed lungs, denser pulmonary arterioles, and larger alveoli (2, 3). Hypoxia could induce epithelial injury, influence alveolar homeostasis, and cause a series of pulmonary diseases, such as pulmonary hypertension (4, 5), chronic obstructive pulmonary disease (6, 7), and pulmonary fibrosis (8). Alveolar type I epithelial (ATI) and alveolar type II epithelial (ATII) cells have covered the alveolar surface. ATII can transform into ATI and is responsible for the lungs' repair, recycling, and production (9, 10). ATII could undergo cell death and replace by myofibroblasts in hypoxia-induced IPF, which prevents the repairing and renewal of the alveolar wall (11). The injury of regeneration and transdifferentiation in alveolar epithelial cells are vital points that lead to the disease under hypoxia-induced, which may result in breaks in epithelial basement membranes of alveoli (9). Activation of endoplasmic reticulum stress (12), a different expression of ROS (13), and hemoglobin (14) could involve in the oxygen-sensing pathway in alveolar epithelial cells. Alternative splicing (AS) is one of the essential mechanisms in post-transcriptional regulation and could be regulated by many biotic and abiotic stress factors, especially tightly associated with hypoxic adaptation of cells (15). For example, splicing targets of alternative first exon usage, exon skipping, and intron retention could potentially contribute to cancer cell hypoxic adaptation by promoting cancer cell proliferation, transcriptional regulation, and migration (16–18). Large-scale alterations in alternative splicing of ribosomal protein mRNAs were influenced by hypoxia (19). Promotes expression of the angiogenesis inhibitory alternatively spliced hypoxia-inducible factor-3α (*HIF*-*3*α*)* IPAS isoform, and *HIF*-*1*α splicing during angiogenesis could be regulated by hypoxia (18, 20). Recent studies have identified alternative splicing events that exist in lung (21–23), heat (24), and ovary (25). Until now, the analysis of alternative splicing in ATII was rarely reported. Here, we carried out a comparative study of AS in ATII during normoxic (21% O_2_) and hypoxic (21% O_2_) to explore the patterns and conservation of AS between Tibetan pigs and Landrace pigs. Our results supported further development of hypoxia-associated splicing events in ATII, representing one of the steps forward in the hypoxic adaptation of Tibetan pigs.

## Materials and methods

### Samples

Alveolar type II epithelial primary cells from newborn male Tibetan pigs and Landrace pigs were isolated and cultured as described previously (26) with minor modifications. ATII cells were collected at 48 h, which were cultured under normoxic conditions (21% O_2_, 5% CO_2_, and 79% N_2_) between Tibetan pigs (TN, *n* = 3) and Landrace pigs (LN, *n* = 3), and under hypoxic conditions (2% O_2_, 5% CO_2_, and 98% N_2_) between Tibetan pigs (TL, *n* = 3) and Landrace pigs (LL, *n* = 3), respectively.

### RNA extraction, library construction, and sequencing

Total RNA was extracted from ATII cells using a TRIzol reagent kit (Invitrogen, Carlsbad, CA, USA), treated, removed, and precipitated using DNase I (NEB, Beijing, China) phenol-chloroform, and ethanol. Total RNA quality was determined using an Agilent 2100 Bioanalyzer (Agilent Technologies, Palo Alto, CA, USA) and checked using Rnase-free agarose gel electrophoresis.

The mRNAs and non-coding RNAs (ncRNAs) were obtained by removing ribosome RNAs (rRNAs) from total RNA, fragmented into short fragments using fragmentation buffer and reverse transcribed into complementary DNA (cDNA) with random primers, and synthesized to second-strand cDNA. Next, the cDNA fragments were ligated to Illumina sequencing adapters by purifying with a QiaQuick PCR extraction kit (Qiagen, Venlo, The Netherlands), and the second-strand cDNA was digested. The twelve cDNA libraries were generated, purified, and sequenced using Illumina HiSeq™ 4000 by Gene Denovo Biotechnology Co. (Guangzhou, China).

### Relative abundance of mRNA

Clean, high-quality reads were obtained and filtered from raw reads using fastp (27) (version 0.18.0) and removing the rRNA mapped reads to the rRNA database. The RefSeq (*Sus scrofa* 11.1) databases were mapped using HISAT2 (28). Transcripts reconstruction was carried out with software Stringtie and HISAT2. HTSeq counted the number of reads aligned to each gene and exon. A fragment per kilobase of transcript per million mapped reads (FPKM) value was calculated to quantify its expression abundance. We carried out differentially expressed genes (DEGs) using a threshold of |log2(fold_change)| ≥2 and a false discovery rate (FDR) adjusted *p*-value <5%.

### Identification of AS types and counts

Paired-end raw data were first evaluated using FastQC v0.11.8 (29), and quality control using the FASTX toolkit to trim bases in 5' sequences and trimmomatic to trim adaptor sequences and low-quality reads (30, 31). High-quality reads were aligned to the reference genome sequence (*Sus scrofa* 11.1) and merged using TopHat2 v2.1.1 (32) and Cufflinks v2.2.1 (33). Differential AS events were identified and analyzed using rMATS (version 4.0.1, http://rnaseq-mats.sourceforge.net/index.html) and AS variations of each transcription region by using StringTie software among four groups. The FDR <0.05 in the comparison was used to identify different AS events. The classification of AS was as follows: alternative 5′ splice sites (A5SSs), alternative 3' splice sites (A3SSs), retained introns (Ris), skipped exons (Ses), and mutually exclusive exons (MXEs) were the main categories of selective splicing.

### Enrichment and integrative analysis of the alternatively spliced DEmRNAs regulatory network

We analyzed alternatively spliced DEGs using the Kyoto Encyclopedia of Genes and Genomes (KEGG) and Gene Ontology (GO) in the online tool database for annotation, visualization, and integrated discovery (DAVID, version 6.7, https://david.ncifcrf.gov/). GO was used to determine and explore the functions of the alternatively spliced DEGs as molecular function, biological process, and cellular component. KEGG analyzed alternatively spliced DEGs to reveal their roles, regulation processes, and enrichment in different biological pathways. The *p*-values <0.05 were considered significantly different enriched biological pathways. The co-expression regulatory network of alternatively spliced DEGs is generated using the PCC, and the diagram only shows the top 250. The potential regulatory network was constructed by Cytoscape (34).

### qRT-PCR validation of AS events

The four groups randomly selected three alternatively spliced DEGs for Real-time quantitative reverse transcription polymerase chain reaction (qRT-PCR) verification. Total RNA was extracted from ATII cells to synthesize cDNA using a FastQuant cDNA first-strand synthesis kit (TianGen, China). The cDNA was subjected to qRT-PCR analysis. Transcript-specific primers (Supplementary Table S1 in Supplementary material 1) were designed based on the unique regions of selected alternatively spliced DEGs using Primer 5.0 software, β*-actin* was used as reference genes, and expression levels were calculated using the 2^−Δ*ΔCt*^ method. PCR conditions were performed as follows: 95°C for 30 s, forty cycles at 95°C for 5 s, 60°C for 30 s, and 72°C for 30 s.

## Results

### Forms of alternative splicing events

**Figure 1:**
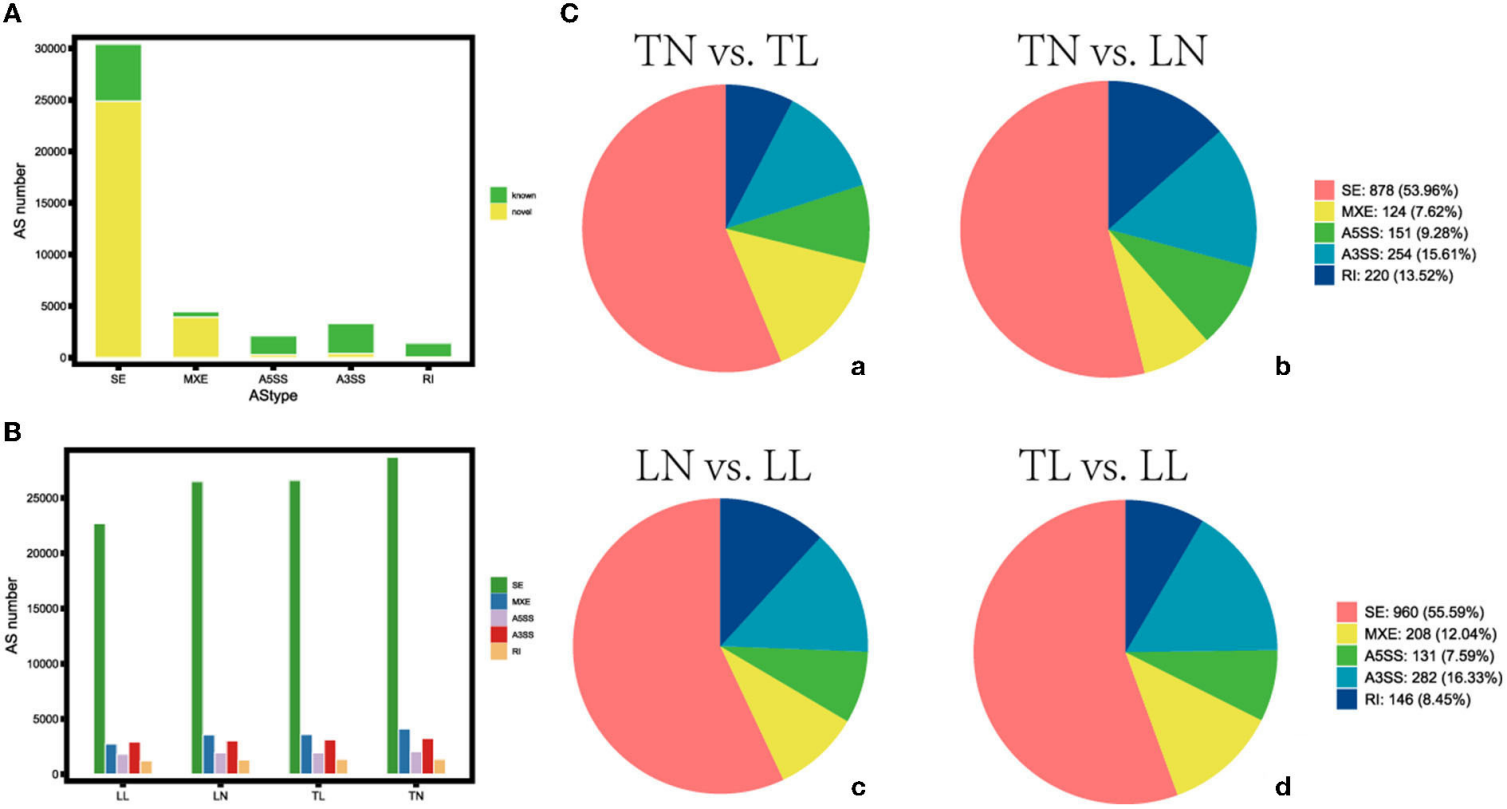
Distribution of the different types of alternative splicing (AS) events. **(A)** Distribution of the known and novel AS. **(B)** The number of AS events among four groups. **(C)** Different types of AS events (a) between TN and TL, (b) between TN and LN, (c) between LN and LL, and (d) between TL and LL groups.

**Figure 2:**
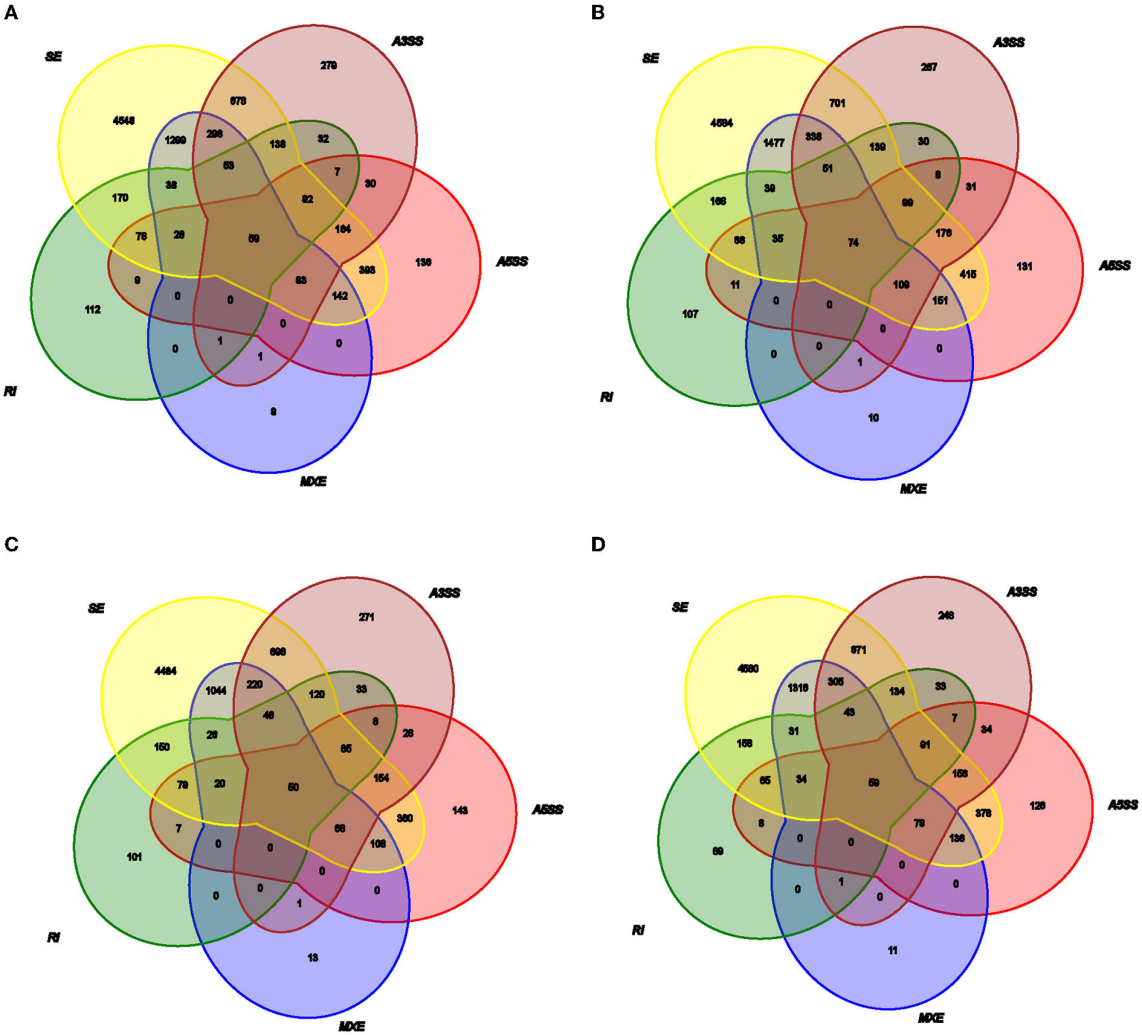
Relationship of the different types of AS events in **(A)** TL, **(B)** TN, **(C)** LL, and **(D)** LN.

The average 11,245,202,725 bp clean data were obtained from 11,516,010,250 bp raw reads after filtering out low-quality data among twelve libraries (Supplementary Table S2 in Supplementary material 1). The 41,642 AS events corresponding to 9,924 genes were identified using genomic information and transcript data from the RNA-seq dataset (Figure 1, Supplementary material 2). Hypoxia-induced generally increased the number of AS events. Therefore, a total of five alternative splicing forms were obtained through data mapping analysis, such as A5SS, MXE, A3SS, SE, and RI, which revealed Ses as the most abundant event type (73.01%), followed by MXE (10.70%), A3SS (7.95%), and A5SS (5.02%); mutually RI occurred in only 3.32% of AS events among the four groups (Figure 2). Furthermore, 1,444, 2,192, 2,522, 954, and 9,238 alternatively spliced genes undergo A5SS, A3SS, MXE, RI, and SE events, respectively. The results demonstrated that almost all DEGs underwent at least one AS event. The frequencies of AS events were similar among different groups. The highest frequency (64) of AS events was in the *TNC* gene (ncbi_397460) in the TN group (Supplementary material 3), and the highest frequency of AS events was in the *OBSCN* gene among the four groups.

### Alternatively, spliced DEGs in ATII cells response to hypoxia

**Figure 3:**
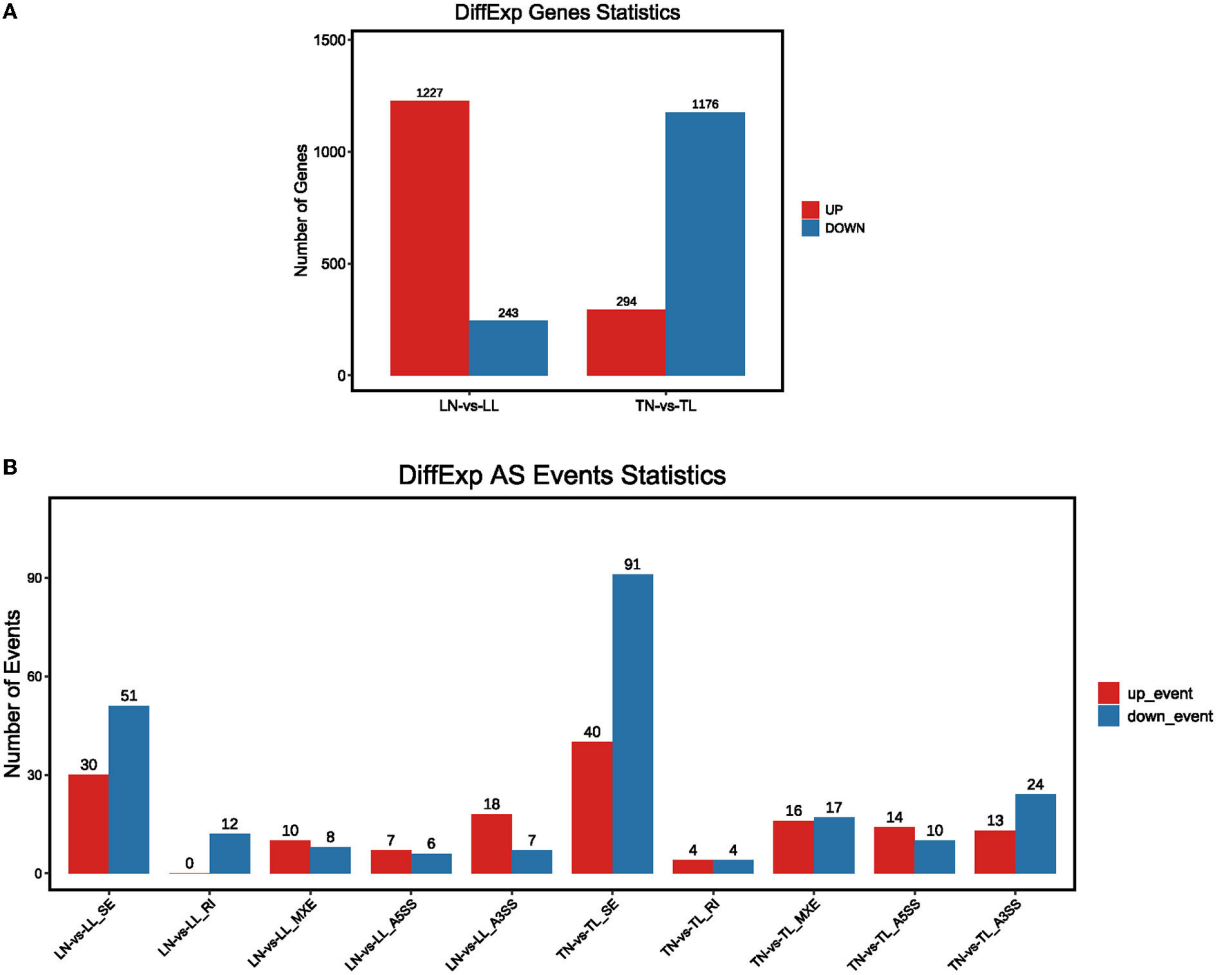
The presence of alternate spliced DEGs in the cross region of normoxic and hypoxic groups. **(A)** DEGs with alternate splicing were distributed in Tibetan pigs and landrace pigs in normoxia and hypoxia groups; **(B)** Distribution of DEGs with alternate splicing in five splicing events of Tibetan pigs and Landrace pigs in normoxia and hypoxia groups, respectively.

The analysis found that most of the DEGs underwent AS events. Approximately, 33,985 AS events of the total expressed genes and 1,763 significant AS events were screened between TN and TL groups (Supplementary Table S3 in Supplementary material 1). We further selected the 1,470 intersection genes between normoxic (21% O_2_, TN and LN) and hypoxic (2% O_2_, TL and LL) groups for significant hypoxia-related genes to identify their AS events, which revealed that 75.00% of them undergo diverse AS (Supplementary material 4). The AS of 901 intersection genes associated with hypoxia, such as *EPAS1, NREP*, and *VPS13B*, were only mediated by one or two events. Another 189 genes, such as *CCDC14, NKTR*, and *ATRX*, exhibited complex AS. For example, AS in *NFAT5, ECM1, ZBTB20, KMT2E, ZMYM1*, and *PLAGL1* were classified as five basic types between normoxic and hypoxic groups. We found that 233 differential splicing events of 4,514 AS circumstances were present in 1,470 differentially expressed intersection genes between the TN and TL groups (Figure 3, Supplementary Table S4 in Supplementary material 1).

### GO and KEGG enrichment of alternatively spliced DEGs

**Figure 4:**
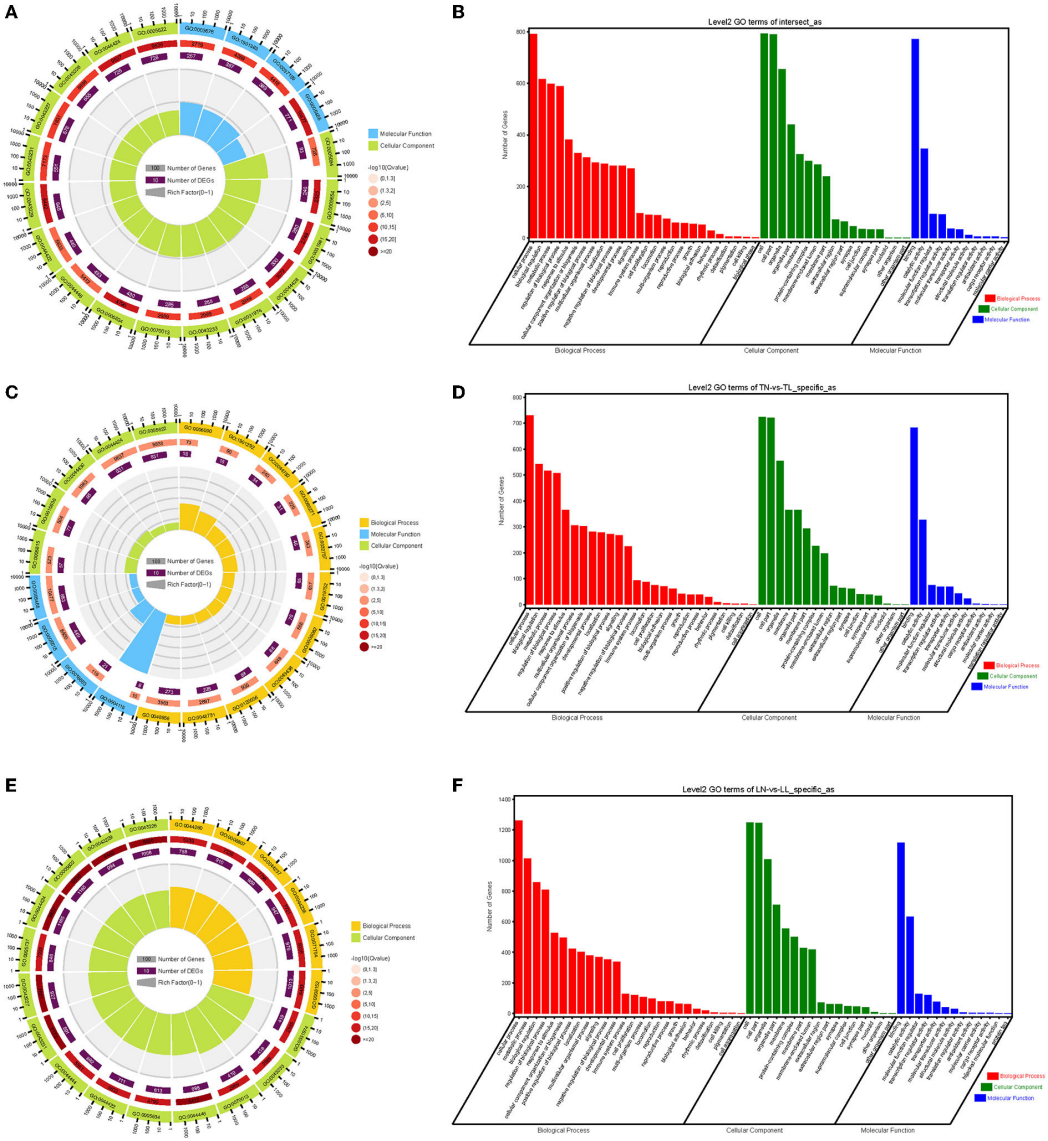
GO functional annotation **(A,B)** between normoxic (TN and LN) and hypoxic (TL and LL) groups, **(C,D)** between TN and TL (excluding alternatively spliced DEGs shared between normoxia and hypoxia groups), and **(E,F)** between LN and LL (excluding alternatively spliced DEGs shared between normoxia and hypoxia groups) groups.

The enrichment analyses of alternatively spliced DEGs were performed by GO analysis to investigate the biological function of AS events between normoxic (21% O_2_, TN and LN) and hypoxic (2% O_2_, TL and LL) groups. The results showed that 817 biological processes, 163 molecular functions, and 114 cellular components were significantly enriched (*p* < 0.05) (Figures 4A,B). For AS genes of DEGs, biological processes were enriched considerably, such as regulation of nucleobase-containing compound metabolic process (GO: 0019219), nucleic acid metabolic process (GO: 0090304), and nucleobase-containing compound metabolic process (GO: 0006139). Several genes were significantly enriched in the nucleus (GO: 0005634), intracellular part (GO: 0044424), and intracellular (GO: 0005622) cellular component. Binding (GO: 0005488), heterocyclic compound binding (GO: 1901363), and organic cyclic compound binding (GO: 0097159) of molecular functions were most significantly enriched. In a comparison of TN and TL (excluding alternatively spliced DEGs shared between normoxic and hypoxic groups), pyruvate metabolic process (GO: 0006090), binding (GO: 0005488), and intracellular part (GO: 0044424) of biological processes, molecular functions, and cellular components were most significantly enriched (Figures 4C,D). In a comparison of LN and LL (excluding alternatively spliced DEGs shared between normoxic and hypoxic groups), cellular metabolic process (GO: 0044237), catalytic activity (GO: 0003824), and intracellular part (GO: 0044424) of biological processes, molecular functions, and cellular components were most significantly enriched (Figures 4E,F).

**Figure 5:**
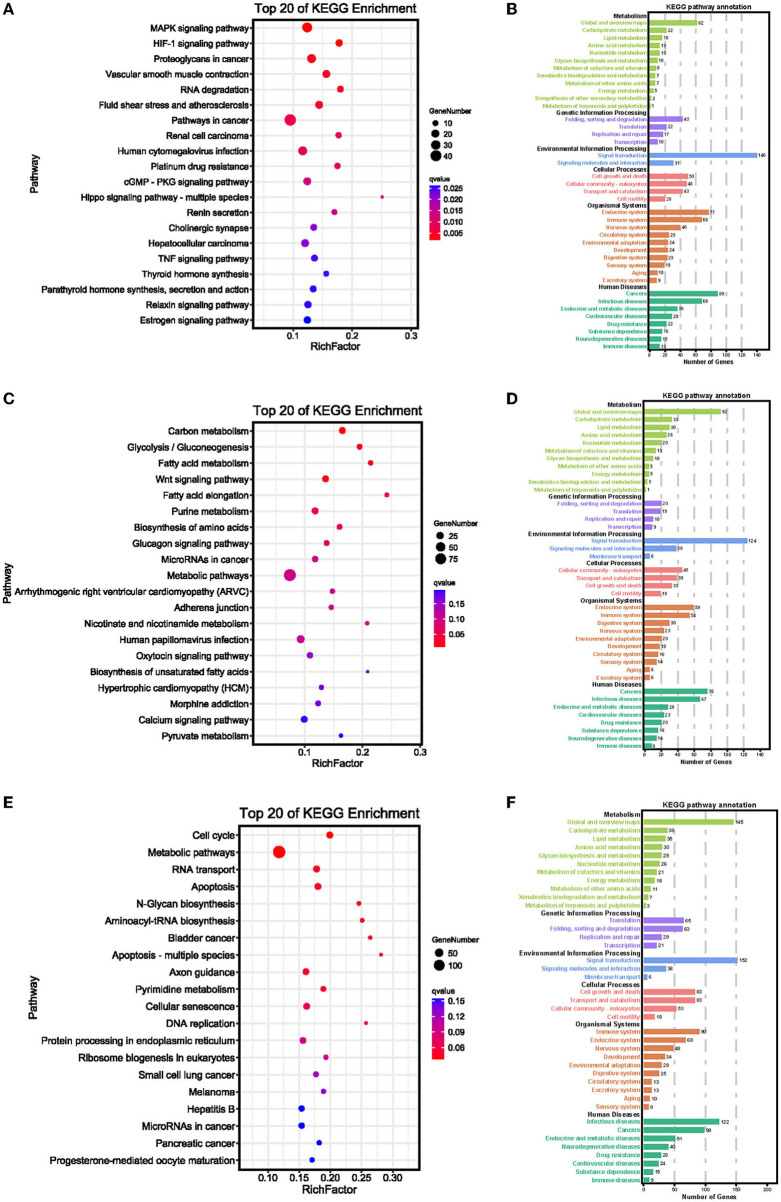
KEGG enrichment pathways of alternatively spliced DEGs **(A,B)** between normoxic (TN and LN) and hypoxic (TL and LL) groups, **(C,D)** between TN and TL (excluding alternatively spliced DEGs shared between normoxia and hypoxia groups), and **(E,F)** between LN and LL (excluding alternatively spliced DEGs shared between normoxia and hypoxia groups) groups. The ordinate is the pathway, and the abscissa is the enrichment factor. Darker colors indicate smaller q-values.

As the AS of mRNAs is directly related to functional characteristics, the function of alternatively spliced DEGs was analyzed by KEGG enrichment. A total of 279 pathways were enriched with 89 pathways significantly enriched (*p* < 0.05), of them MAPK signaling pathway (ko04010), HIF-1 signaling pathway (ko04066), and proteoglycans in cancer (ko05205) were most significantly enriched between normoxic (21% O_2_, TN *vs*. LN) and hypoxic (2% O_2_, TL *vs*. LL) groups (Figures 5A,B). When TL was compared with TN (excluding alternatively spliced DEGs shared between normoxic and hypoxic groups) groups, alternatively spliced DEGs were found to be significantly enriched in carbon metabolism (ko01200), glycolysis/gluconeogenesis (ko00010), and fatty acid metabolism (ko01212) pathways (Figures 5C,D). Cell cycle (ko04110), metabolic pathways (ko01100), and RNA transport (ko03013) were most significantly enriched by abundant genes between LN and LL groups (excluding alternatively spliced DEGs shared between normoxic and hypoxic groups) (Figures 5E,F).

### Coexpression network of alternatively spliced DEGs expression profiles

**Figure 6:**
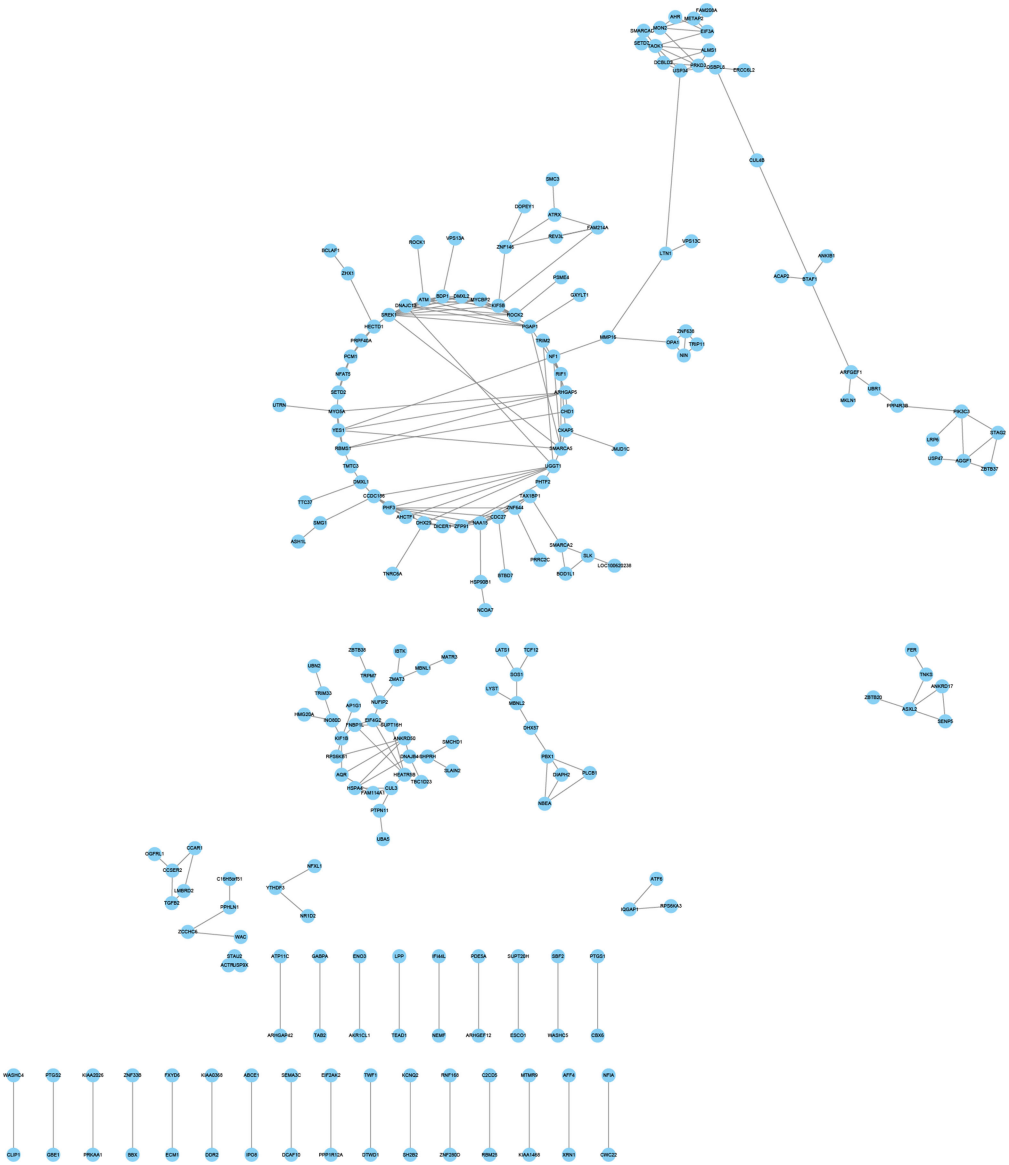
Coexpression network analyses of alternatively spliced DEGs between normoxic (TN and LN) and hypoxic (TL and LL) groups.

Three hypoxia-related co-expression networks of alternatively spliced DEGs were constructed. The top 250 relationship pair network diagrams are listed, such as comparison groups of normoxia and hypoxia, TN and TL (excluding alternatively spliced DEGs shared between normoxia and hypoxia groups), LN and LL (excluding alternatively spliced DEGs shared between normoxia and hypoxia groups) (Figure 6, Supplementary Figures S1, S2). The intersection of comparisons between normoxic (TN, LN) and hypoxic (TL, LL) represented the main differences of ATII cells at different oxygen concentrations gradient. *ROCK2* (ncbi_397445), *KIF5B* (ncbi_595132), and *ZFP91* (ncbi_100525558) were selected as the most affected mRNAs, and there were strong correlations with several RNAs undergoing AS events between normoxic (TN, LN) and hypoxia (TL, LL) groups. Interestingly, *VCAN* (ncbi_397328), *HSD3B1* (ncbi_445539), and *FAM13C* (ncbi_100525364) were most significantly correlated with a large number of alternatively spliced DEGs between TN and TL groups (excluding alternatively spliced DEGs shared between normoxia and hypoxia groups). Meanwhile, *ITGAV* (ncbi_397285), *ADAM9* (ncbi_397344), and *MYOF* (ncbi_100154898) were most significantly correlated with a large number of alternatively spliced DEGs between LN and LL groups (excluding alternatively spliced DEGs shared between normoxia and hypoxia groups).

### Verification of transcripts expression and AS events

Three alternatively spliced DEGs were randomly selected to further test the accuracy of RNA-seq data using qRT-PCR. *HP1BP3, NECTIN2*, and *DDX11* were predicted and identified as having two transcripts, and the type of alternative splicing is SE. The expression levels of the transcript with inclusion and skipping are higher than that of skipping transcript among four groups (Supplementary Figure S3), indicating that the alternative splicing prediction based on RNA-seq data was reliable.

## Discussion

The identification, characterization, and post-transcriptional regulation of alternatively spliced DEGs were widely studied by attracting the interest of researchers (15, 35, 36), such as Xiang pig gilts, bovine, and human. Animals have a more complicated and more extensive intron than plants (37). Transcriptome survey reveals increased complexity of the alternative splicing landscape in Arabidopsis (37–39), and their most common AS events were exon skipping and intron retention, respectively (40, 41). A5SS, MXE, A3SS, SE, and RI were components of five essential AS forms in our study, and this distribution pattern is also similar to that of other animals reported previously (15, 35, 42, 43), indicated that animals might possess similar alternative splicing forms. Alternative splicing events were numerous occur during organ development, tissue maturation, and cell differentiation, suggesting that alternative splicing supports proper development (15). The phenotype may be influenced by modification of gene transcription or translation induced by a hypoxia condition (44). SE may be the primary source of proteomic and transcriptomic and plays a significant role in hypoxia response by regulating genes and determining phenotype as the most abundant event types (73.01%) in ATII cells among four groups (45, 46).

### Regulation of AS in ATII cells response to hypoxia

According to the KEGG enrichment, a total of 1,088 alternatively spliced DEmRNAs were enriched in 279 pathways between normoxic (21% O_2_, TN and LN) and hypoxic (2% O_2_, TL and LL) groups, of which 35, 17, and 25 genes were enriched in MAPK signaling pathway, HIF-1 signaling pathway, and proteoglycans in cancer. MAPK may arise and upregulate the transcription of anti-apoptotic genes under exposure to hypoxia and play critical roles in opposing the inflammatory response and regulating cell proliferation, differentiation, and apoptosis, which may be a novel strategy for the treatment of chronic obstructive pulmonary fibrosis (6, 47–50). Insulin-like growth factors (*IGF1* and *IGF2*) enriched in 30 pathways and underwent one type of AS event between normoxic and hypoxic groups and might act as cross-talk between MAPK pathways and HIF signaling pathway (51), which may reduce ATII cell apoptosis under hypoxic conditions (52). The increase of HIF-1 transcriptional activity under a hypoxia environment is due to a decrease of cellular NAD+, which downregulates Sirt1 to enhance HIF-1α acetylation (53). As expected, we also discovered that several glycolysis-related genes (such as *SLC2A1, HK1, HK2, ENO3*, and *PFKFB3*) undergo one or two AS event types. The frequency of *HK2* was higher in normoxia than that of hypoxia groups, and the frequency of SE events in *ENO3* was lower in LN groups than any others. The frequency of SE events in *HK1* was lowest in LL groups, enriched in the HIF-1 signaling pathway, and may promote anaerobic metabolism by elevating interstitial pressure and alleviating cell damage through glucose metabolism under hypoxia conditions (54, 55). The energy and metabolic intermediates produced through cells rely on glycolysis by hypoxia availability. *HK1* and *HK2*, responsible for the initial steps of glycolysis, convert glucose to glucose-6-phosphate (G-6-P) through phosphorylation, initiating glycolysis and producing pyruvate and lactic acid as energy sources (56, 57). PFKFB enzymes catalyze the synthesis of fructose-2,6-bisphosphate (F-2,6-P2) as one of the numerous glycolytic regulators. *PFKFB3* plays a dominant role in vascular cells, leukocytes, and many transformed cells and catalyzes the conversion of fructose-6-phosphate to fructose-1,6-biphosphate as the number of the four isoforms of PFKFBs (58, 59). *PFKFB3* undergoes SE events and has a lower frequency in LN groups than in any other groups, may control the steady-state concentration of F-2,6-P2, and glycolysis also mediated the generation of growth factors and proinflammatory cytokines in ATII cells under hypoxia condition (59, 60). Thus, glycolysis intermediates can be increased in the intracellular levels of glucose and quickly diverted to anabolic pathways under hypoxia as substrates for lipid and protein biosynthesis and DNA replication to rapidly proliferate ATII cells (61, 62).

*ROCK2, KIF5B*, and Zinc finger protein 91 (*ZFP91*) regulated several mRNAs under hypoxia conditions stimulation as essential hypoxia-inducible genes between normoxia and hypoxia groups. Under hypoxia conditions, pulmonary arterial endothelial cells' proliferation and cell cycle *via* activation of the ROCK2 signaling pathway (63). Cell migration of macrophages and bladder cancer cells may inhibit *ROCK2* expression (64, 65). *ZFP91* could upregulate the expression of *HIF-1*α *via* binds to its promoter region and is involved in various biological processes (66, 67). In summary, the present study shows that the A3SS and SE AS events of *ZFP91* and higher frequency of SE events in *ROCK2* and under normoxia (LN and TN) groups may influence proliferation, apoptosis, and epithelial–mesenchymal transition of ATII cells (63–67).

### Functional effects of alternatively spliced DEGs of Tibetan pigs and landrace pigs at hypoxia conditions

Although the alternatively spliced DEGs in the same oxygen concentration of Tibetan pigs and Landrace pigs should have similar alternative splicing, 18, 12, and 10 alternatively spliced DEGs were most significantly enriched in carbon metabolism, glycolysis/gluconeogenesis, and fatty acid metabolism among the TN and TL groups (excluding alternatively spliced DEGs shared between normoxic and hypoxic groups). *LDHA* undergoes SE and MXE events and is significantly enriched in the glycolysis/gluconeogenesis pathway of ATII cells in Tibetan pigs under normoxia and hypoxia, a net charge of −6, and preferentially converts pyruvate to lactate, and occupies plasma membrane and mitochondrial with *LDHB* isoforms (68). Previous research reveals that *CD36* and intracellular lipid expression and content were augmented in hypoxic hepatocytes. The membrane-bound sterol regulatory element-binding protein (*SREBP*) transcription factors could respond to lipid availability and regulate lipid uptake and synthesis as central regulators of lipid homeostasis (69). Fatty acids were identified as a physiological modulator of HIF and have similar functions to oxygen, defining a mechanism for lipoprotein regulation (70). Several alternatively spliced DEGs, such as *ACADL, EHHADH*, and *CPT1A*, undergo one or two AS types of ATII cells, and different types and frequencies of AS event may be a constant supply of lipids were obtained either from the circulation or *de novo* synthesis for ATII cells growth better under hypoxia condition (71, 72).

In contrast to the results obtained from the comparison of ATII cells of Landrace pigs under normoxic and hypoxic conditions, the pathways related to the alternatively spliced DEGs identified from the comparison between LN and LL (excluding alternatively spliced DEGs shared between normoxia and hypoxia groups) were associated with cell cycle, metabolic pathways, RNA transport, and apoptosis. Available evidence suggests hypoxia compensates for cell cycle arrest with decreased S-phase entry in mature ECs and progenitor differentiation during angiogenesis (73). The *p53* is a cell cycle regulator and apoptosis in the white shrimp in response to hypoxia (74), and the miR-493-STMN-1 pathway could promote hypoxia-induced epithelial cell cycle arrest in G_2_/M phase (75). *CDK2* (cyclin-dependent kinase 2) undergo AS event between LN and LL groups, which could be activated by either *CCNE* (cyclin E) or *CCNA* (cyclin A) at the G1/S phase transition or S phase, and mediates degradation of *HIF*-*1*α at the G1/S change (76). *MCM7* and *MCM3* undergo AS events between LN and LL (excluding alternatively spliced DEGs shared between normoxia and hypoxia groups) groups, bind to the amino-terminal PER-SIM-ARNT (PAS) domain, and the carboxyl terminus of *HIF-1*α to maintain their stability (77).

## Conclusion

In this study, we disclosed features of AS events in ATII cells through RNA-seq data. The results indicated that different types of AS and regulatory networks might partially contribute to the significant variance in ATII cells of Tibetan pigs and Landrace pigs under different oxygen concentrations. *ACADL, EHHADH*, and *CPT1A* may be a constant supply of lipids were obtained either from the circulation or *de novo* synthesis for ATII cells of Tibetan pigs growth better under hypoxia conditions. Therefore, this study provided a better understanding of the effects of different AS of candidate functional genes on ATII cells' response to hypoxia.

## Data availability statement

The datasets presented in this study can be found in online repositories. The names of the repository/repositories and accession number(s) can be found in the article/Supplementary material.

## Ethics statement

The animal study was reviewed and approved by Livestock Care Committee of Gansu Agricultural University.

## Author contributions

SZ and YY were the overall project leader who provided financial support and experimental conception. HY was involved in data analyses, statistical analyses, language revisions, journal selection, and manuscript submissions and revisions. XL and ZW contributed to the experimental design and implementation. CG contributed to the supervision and assistance of students in managing animals and collecting and analyzing samples. YL and YR were responsible for the trial implementation, supervision of students collecting and analyzing samples, and manuscript preparation. YC and TJ contributed to supervision of sample collection and analysis and manuscript editing. All authors contributed to the article and approved the submitted version.

## Funding

The study was supported by the National Natural Science Foundation of China (32060730).

## Conflict of interest

The authors declare that the research was conducted in the absence of any commercial or financial relationships that could be construed as a potential conflict of interest.

## Publisher's note

All claims expressed in this article are solely those of the authors and do not necessarily represent those of their affiliated organizations, or those of the publisher, the editors and the reviewers. Any product that may be evaluated in this article, or claim that may be made by its manufacturer, is not guaranteed or endorsed by the publisher.

## Supplementary material

Supplementary Material 1

Supplementary Table S1 Primers used to detect alternatively spliced differentially expressed genens (DEGs) in ATII cells of pigs by qRT-PCR.

Supplementary Table S2 Overview of the reads and quality filtering of mRNA libraries.

Supplementary Table S3 The number of genes underwent alternative splicing (AS) events.

Supplementary Table S4 AS events of Tibetan pigs and Landrace pigs were present in DEGs between normoxic and hypoxic groups.

Supplementary Material 2

2.1. The A3SS events corresponding to genes were identified.

2.2. The A5SS events corresponding to genes were identified.

2.3. The MXE events corresponding to genes were identified.

2.4. The RI events corresponding to genes were identified.

2.5. The SE events corresponding to genes were identified.

Supplementary Material 3

3.1. The frequency of AS events in the TN group.

3.2. The frequency of AS events in the TL group.

3.3. The frequency of AS events in the LN group.

3.4. The frequency of AS events in the LL group.

Supplementary Material 4

4.1. Differentially expressed genes between normoxic (21% O_2_, TN and LN) and hypoxic (2% O_2_, TL and LL) groups.

4.2. Alternatively spliced DEGs between normoxic (21% O_2_, TN and LN) and hypoxic (2% O_2_, TL and LL) groups.

Supplementary Material 5

Supplementary Figure S1 Coexpression network analyses of alternatively spliced differentially expressed genes (DEGs) between TN and TL (excluding alternatively spliced DEGs shared between normoxia and hypoxia groups) groups.

Supplementary Figure S2 Coexpression network analyses of alternatively spliced DEGs between LN and LL (excluding alternatively spliced DEGs shared between normoxia and hypoxia groups) groups.

Supplementary Figure S3 **(A)** Expression patterns of three alternatively spliced DEGs. **(B)** Expression patterns of eight randomly selected DEGs. Histogram represents the change in transcript level according to the FPKM value of RNA-seq (left y-axis), and broken line indicates that relative expression level defense by RT-PCR (right y-axis).

## Abbreviations

DEGs: differentially expressed genes
TN: ATII cells of Tibetan pigs were cultured under 21% O_2_
TL: ATII cells of Tibetan pigs were cultured under 2% O_2_
LN: ATII cells of Landrace pigs were cultured under 21% O_2_
LL: ATII cells of Landrace pigs were cultured under 2% O_2_.
