# Supplementary material for: Alternative splicing signature of alveolar type II epithelial cells of Tibetan pigs under hypoxia-induced
Source: Front Vet Sci. 2022 Sep 16;9:984703. doi: 10.3389/fvets.2022.984703 (PMC9523697; doi:10.3389/fvets.2022.984703)
Supplement: Supplementary Material 1 [file Data_Sheet_1.doc]

Table S1 Primers used to detect alternatively spliced DEGs in ATII cells of pigs by qRT-PCR

| Genes | Primer sequences (5'-3') | Annealing temperature/℃ |
| --- | --- | --- |
| *HP1BP3-2* | F: TGAGAAGCTGTGCGGGACG | 60  60 |
|  | R: TACGAATCGGCATGGTGT |  |
| *HP1BP3-1* | F: GCAGAGGCTTCAGAGTGT | 60  60 |
|  | R: GAATCGGCATGGTGTTAT |  |
| *NECTIN2-2* | F: TCTGCACGGTCACCAACG | 60  60 |
|  | R: GGCGGCTTGTATGAGGGAG |  |
| *NECTIN2-1* | F: CAGCGGAAGGAGCAGAGGCT | 60  60 |
|  | R: TCGGCGGCTTGTATGAGGG |  |
| *DDX11-2* | F: TAGCACTGGCTGGGAATT | 60  60 |
|  | R: TCTGGATGGGATAGGGTG |  |
| *DDX11-1* | F: GCCTACACCACAGCCACA | 60 |
|  | R: CTCAAATATCCCAATCTTGC |  |
| *β-actin* | F: CAGTCGGTTGGATGGAGCAT  R: AGGCAGGGACTTCCTGTAAC | 60 |

Table S2 Overview of the reads and quality filtering of mRNA libraries

| Sample | RawDatas | CleanData(%) | AF_Q20 | AF_Q30 | AF_GC |
| --- | --- | --- | --- | --- | --- |
| LL-1 | 62403492 | 60866934 (97.54%) | 9173313324 | 8811997105 | 4236573072 |
| LL-2 | 25633118 | 24508662 (95.61%) | 3777680765 | 3645029380 | 1735618420 |
| LL-3 | 46503574 | 44840052 (96.42%) | 6834630271 | 6581418310 | 3208746606 |
| LN-1 | 61799310 | 59986428 (97.07%) | 9070593725 | 8712775720 | 4372610179 |
| LN-2 | 63897660 | 62162356 (97.28%) | 9362285143 | 8974106859 | 4551749810 |
| LN-3 | 67556926 | 65719906 (97.28%) | 9913641106 | 9516406381 | 4758709867 |
| TL-1 | 102037632 | 99724036 (97.73%) | 14973512308 | 14379653290 | 7259467329 |
| TL-2 | 98882120 | 96772832 (97.87%) | 14520839322 | 13949795079 | 6974155924 |
| TL-3 | 91099604 | 89034416 (97.73%) | 13371144377 | 12820447271 | 6411590130 |
| TN-1 | 117092830 | 115082170 (98.28%) | 17151172274 | 16457397257 | 8313005466 |
| TN-2 | 89798010 | 88022768 (98.02%) | 13142387754 | 12587436052 | 6336147586 |
| TN-3 | 94576544 | 92895658 (98.22%) | 13880053597 | 13357991075 | 6687507426 |

Table S3 The number of genes underwent AS events.

|  | **TN vs. TL** | | **LN vs. LL** | | **LN vs. TN** | | **LL vs. TL** | |
| --- | --- | --- | --- | --- | --- | --- | --- | --- |
| Event  Types | Total events | Significant events | Total events | Significant events | Total events | Significant events | Total events | Significant events |
| SE | 24568 | 993 | 17594 | 782 | 24453 | 878 | 17718 | 960 |
| MXE | 3245 | 261 | 1934 | 130 | 3227 | 124 | 1932 | 208 |
| A5SS | 1871 | 155 | 1581 | 108 | 1830 | 151 | 1606 | 131 |
| A3SS | 3026 | 219 | 2581 | 189 | 2905 | 254 | 2658 | 282 |
| RI | 1275 | 135 | 1096 | 162 | 1221 | 220 | 1126 | 146 |
| Total | 33985 | 1763 | 24786 | 1371 | 33636 | 1627 | 25040 | 1727 |

Table S4 AS events of Tibetan pigs and Landrace pigs were present in DEGs between normoxic and hypoxic groups

|  | LN-vs-LL | | | | TN-vs-TL | | | |
| --- | --- | --- | --- | --- | --- | --- | --- | --- |
| Type | all_event | diff_event | up_event | down_event | all_event | diff_event | up_event | down_event |
| SE | 1994 | 81 | 30 | 51 | 3329 | 131 | 40 | 91 |
| RI | 92 | 12 | 0 | 12 | 107 | 8 | 4 | 4 |
| MXE | 208 | 18 | 10 | 8 | 431 | 33 | 16 | 17 |
| A5SS | 193 | 13 | 7 | 6 | 236 | 24 | 14 | 10 |
| A3SS | 346 | 25 | 18 | 7 | 411 | 37 | 13 | 24 |
