# Supplementary material for: Alternative splicing signature of alveolar type II epithelial cells of Tibetan pigs under hypoxia-induced
Source: Front Vet Sci. 2022 Sep 16;9:984703. doi: 10.3389/fvets.2022.984703 (PMC9523697; doi:10.3389/fvets.2022.984703)
Supplement: Supplementary Material 5 [file Data_Sheet_2.doc]

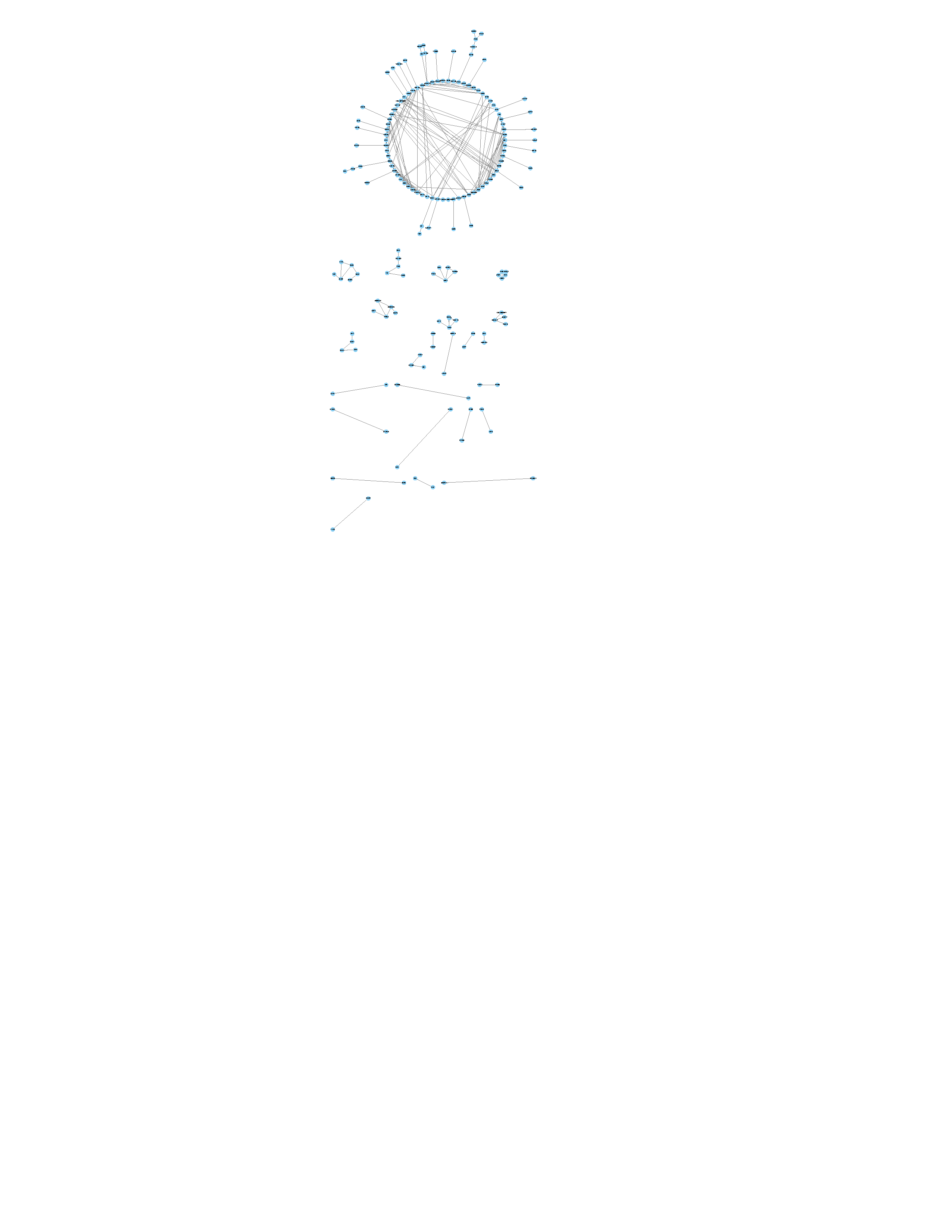


Figure S1 Coexpression network analyses of alternatively spliced DEGs between TN and TL (excluding alternatively spliced DEGs shared between normoxia and hypoxia groups) groups.


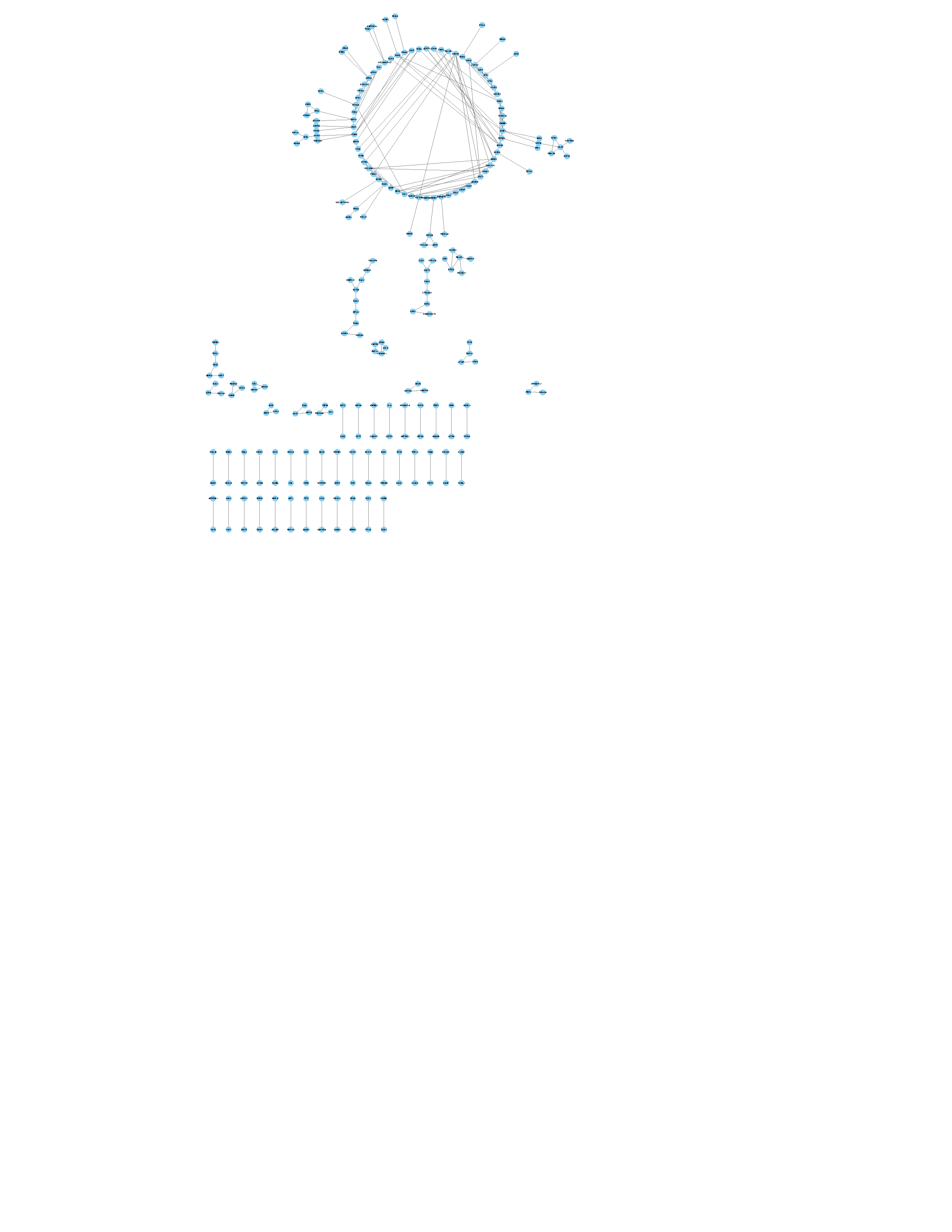


Figure S2 Coexpression network analyses of alternatively spliced DEGs between LN and LL (excluding alternatively spliced DEGs shared between normoxia and hypoxia groups) groups.


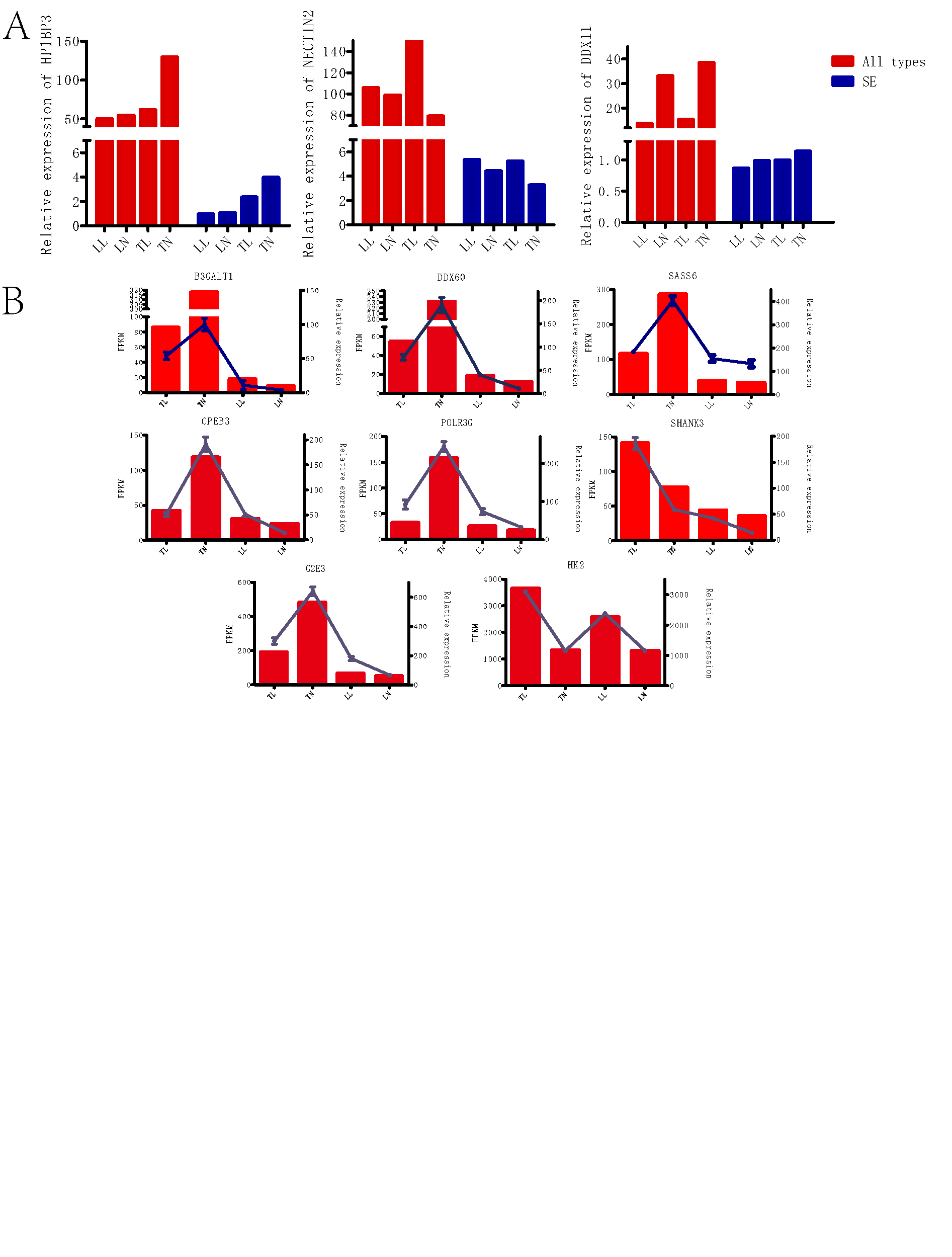


Figure S3 A Expression patterns of eight randomly selected DEGs. Histogram represent the change in transcript level according to the FPKM value of RNA-seq (left y-axis), and Broken line indicate that relative expression level defense by RT-PCR (right y-axis). B. Expression patterns of three alternatively spliced DEGs.
